# DDX58 deficiency leads to triple negative breast cancer chemotherapy resistance by inhibiting Type I IFN-mediated signalling apoptosis

**DOI:** 10.3389/fonc.2024.1356778

**Published:** 2024-03-14

**Authors:** Shiyu Cao, Xinyi Long, Lin Xiao, Peichuan Zhang, Mengjia Shen, Fei Chen, Chunjuan Bao, Xiaorong Zhong, Ting Luo, Feng Ye

**Affiliations:** ^1^ Department of Pathology and Institute of Clinical Pathology, Frontiers Science Center for Disease-related Molecular Network, West China Hospital, Sichuan University, Chengdu, China; ^2^ Institute for Breast Health Medicine, Cancer Center, Breast Center, West China Hospital, Sichuan University, Chengdu, Sichuan, China

**Keywords:** TNBC, DDX58, chemotherapy resistance, Type I IFN, apoptosis

## Abstract

**Introduction:**

Triple-negative breast cancer (TNBC) is characterized by its aggressive nature and absence of specific therapeutic targets, necessitating the reliance on chemotherapy as the primary treatment modality. However, the drug resistance poses a significant challenge in the management of TNBC. In this study, we investigated the role of *DDX58* (DExD/H-box helicase 58), also known as RIG-I, in TNBC chemoresistance.

**Methods:**

The relationship between *DDX58* expression and breast cancer prognosis was investigated by online clinical databases and confirmed by immunohistochemistry analysis. *DDX58* was knockout by CRISPR-Cas9 system (DDX58-KO), knockdown by DDX58-siRNA (DDX58-KD), and stably over expressed (DDX58-OE) by lentivirus. Western blotting, immunofluorescence and qPCR were used for related molecules detection. Apoptosis was analyzed through flow cytometry (Annexin V/7AAD apoptosis assay) and Caspase 3/7 activity assay.

**Results:**

Patients with lower expression of *DDX58* led to lower rate of pathological complete response (pCR) and worse prognosis by online databases and hospital clinical data. DDX58-KD cells showed multiple chemo-drugs resistance (paclitaxel, doxorubicin, 5-fluorouracil) in TNBC cell lines. Similarly, DDX58-KO cells also showed multiple chemo-drugs resistance in a dosage-dependent manner. In the CDX model, tumours in the DDX58-KO group had a 25% reduction in the tumour growth inhibition rate (IR) compared to wild-type (WT) group after doxorubicin (Dox) treatment. The depletion of *DDX58* inhibited proliferation and promoted the migration and invasion in MDA-MB-231 cells. The findings of our research indicated that DDX58-KO cells exhibit a reduction in Dox-induced apoptosis both in vivo and in vitro. Mechanistically, Dox treatment leads to a significant increase in the expression of double-stranded RNAs (dsRNAs) and activates the DDX58-Type I interferon (IFN) signaling pathway, ultimately promoting apoptosis in TNBC cells.

**Discussion:**

In the process of TNBC chemotherapy, the deficiency of *DDX58* can inhibit Dox-induced apoptosis, revealing a new pathway of chemotherapy resistance, and providing a possibility for developing personalized treatment strategies based on *DDX58* expression levels.

## Introduction

The *DDX58* (DExD/H-box helicase 58) gene encodes a cytosolic receptor called RIG-I (retinoic acid-inducible gene I) and belongs to the RIG-I-like receptor (RLR) family (1). *DDX58*, *MDA5* (melanoma differentiation Factor 5) and *LGP2* (laboratory of genetics and physiology 2) are three central members of the RLR family (2). *DDX58* could recognize double-stranded viral RNAs containing two or three 50-phosphatesd physically, associate with viral RNA, and undergo conformational changes that activate downstream signalling molecules, including the essential adaptor *MAVS* (also known as *IPS-1*) (3–6). This association ultimately leads to the activation of the master transcription factors IRF3/IRF7, resulting in the production of Type I IFNs and other inflammatory cytokines that play a crucial role in combating viral infections (7–9).

Some recent studies demonstrate that *DDX58* plays a critical role in promoting cell death in several types of cancer cells. In melanoma, *DDX58* activation induces massive cancer cell apoptosis by synergistic with Bcl2 silencing, and this process depends on the expression of IFN (10). And in breast tumours, the activation of *DDX58* could decrease tumour growth and metastasis, and the synthetic RIG-I agonist can induce breast tumour cell death *in vivo* (11). It is reported that, the activation of DDX58 stimulates cell apoptotic signalling pathways and inhibits the growth of xenografts in human gastric adenocarcinoma (12). Similarly, in human head and neck squamous cell carcinoma, higher level of DDX58 activation also leads to cell apopotosis (13). Furthermore, Type I IFNs signalling, which is the downstream of DDX58, is also reported influencing cellular differentiation, proliferation and apoptosis (14). By promoting the apoptosis of neoplastic cells, the antitumour activity of Type I IFNs can be accomplished (15). And apoptosis induced by Type I IFN activation is also reported in glioblastoma (16). All these results indicate that activation of DDX58 and the downstream Type I IFN pathway play essential roles in mediating viral infections, prohibiting proliferation, as well as promoting apoptosis.

Moreover, growing evidence indicates that Type I IFN signalling is involved in the success of conventional chemotherapeutics, radiotherapy and immunotherapy (17). Antonella et al. describe the contribution of Type I interferon signalling to chemotherapy efficacy in cancer cells (18). Type I IFN signalling is also essential for increasing the therapeutic effects of immunotherapies (19–21). Various types of cells were reported be engineered to express Type I IFNs to enhance the tumour-killing ability of immune effector cells from the host (22–24). Some studies report that radiation therapy could induce the production of Type I IFN signalling in tumour cells (25, 26). Although activation of Type I IFN signalling has been repeatedly reported in radiotherapy and chemotherapy-induced cell death, this process mediated by *DDX58* activation has only been reported in radiotherapy (27, 28). Up to now, little was known about whether *DDX58* activation mediated by Type I IFN signalling was involved in increasing therapeutic effects of chemotherapy.

The latest International Agency for Research on Cancer (IARC) report showed that there were 2.26 million new cases of breast cancer in women worldwide, accounting for almost 1/4 of the total number of new cancers, far exceeding other malignant tumours (29). Triple-negative breast cancer (TNBC) has the highest rate of early local recurrences and distant metastases, leading to the worst prognosis compared to other subtypes (30). TNBC accounts for 10–15% of all breast cancers (31), and the mainstay of treatment is still chemotherapy (32). Approximately 30%-40% of cases will develop distant metastasis, which leads to poor clinical outcomes (33–36). Despite significant efforts to elucidating the mechanisms of TNBC chemoresistance, there is still a lack of effective strategies to overcome chemotherapy resistance in TNBC.

A combination of chemotherapy that relies on paclitaxel or doxorubicin is the basic regimen for TNBC therapy. However, the mechanism of their drug resistance has not been clearly revealed. In this study, we first found that low expression of DDX58 associated with poor prognosis and worse chemotherapy response in TNBC. Then, we focused on exploring the mechanism of *DDX58* in chemotherapy resistance. Interestingly, we found that the *DDX58* signalling pathway was activated by the accumulation of dsRNAs under Dox treatment. These molecular events triggered cell apoptosis mediated by *DDX58*-Type I IFN signalling pathway in TNBC. The knockout of *DDX58* inhibited cell apoptosis and resulted in Dox chemotherapy resistance in both *in vivo* and *in vitro* models. In conclusion, these results indicate that *DDX58* activity can enhance the effectiveness of Dox treatment for TNBC, suggesting a potential therapeutic target for TNBC treatment.

## Materials and methods

### Cell culture

The cell lines used in this study were all offered by the Department of Pathology, West China Hospital. A panel of 5 breast cancer cell lines were used. And all cell lines were verified by short-tandem repeat (STR) analysis. The cancer cell lines consisted of all the main molecular subtypes of breast cancer, including three TNBC cell lines (MDA-MB-231, MDA-MB-468, BT-549), one Luminal cell line (MCF-7), and one HER2+ cell line (SKBR3). The SKBR3 cell line was cultured in McCoy’s 5A medium (Gibco) supplemented with 10% Fetal Bovine Serum (FBS) (Invitrogen Life Technologies) and 1% penicillin/streptomycin (Invitrogen Life Technologies), while the other cell lines were cultured in RPMI 1640 medium (Gibco) supplemented with 10% FBS and 1% penicillin/streptomycin. All cell lines were maintained by continuous passaging at 37°C with a humidified atmosphere of 5% CO_2_.

### CRISPR/cas9 gene editing system

To generate gene-edited cells, the PX330 plasmid was first linearized with BbsI (NEB) restriction enzyme. The DDX58-sgRNA oligos were then synthesized and cloned into the px330 plasmids using standard molecular cloning techniques. The DDX58-sgRNA sequences used in this study were sgRNA1: 5’GGATTATATCCGGAAGACCC3’ and sgRNA2: 5’ TCTCGGCGGAAAACAATTGA 3’. For transfection, MDA-MB-231 cells were plated at a density of 5x10^5^ cells per well in a six-well plate and allowed to attach overnight. The following day, the cells were transfected with 2 μg of sgRNA1-px330 and 2 µg of sgRNA2-px330 plasmid DNA using lipo3000 transfection reagent (Thermo Fisher Scientific) following the manufacturer’s protocol. After 48 hours, cells were dissociated into single cells and counted using a hemocytometer. To reduce the likelihood of multiple cells being present in a single well, the cells were serially diluted in cell medium to a final concentration of 0.5 cells per 100 μl. A multichannel pipette was then used to distribute 100 μl of diluted cells to each well of a 96-well plate. The 96-well plates were then examined under a microscope, and wells that might have been seeded with multiple cells were marked off after 3-4 days of culturing. The cells were then returned to the 37°C incubator and allowed to expand for 2-3 weeks before being transferred to 48- and 24-well plates. Genomic DNA was extracted from the cells, and PCR was performed to identify positive clones. Genomic DNA of cells was extracted, and polymerase chain reaction (PCR) was used to identify the positive clones. Finally, the positive clones were verified by first-generation sequencing and western blotting.

### RNA interference and transfection

For siRNA transfection, approximately 3 x 10^5^ cells were seeded in six-well tissue culture plates. After 24 hours, when the cells reached a confluence of approximately 70%, they were transfected with control siRNA and DDX58-targeting siRNA using Lipofectamine 3000, following the manufacturer’s protocol. After 48 hours, the cells were lysed, and cell proteins were extracted to validate the knockdown effect of the siRNA. Validated cells were subsequently used for cell proliferation analysis.

### CDX model experiment

All animal experiments were approved and performed in accordance with the West China Hospital of Sichuan University Biomedical Research Ethics Committee (2022-1056). And our study was reported in accordance with ARRIVE guidelines. All mice were raised in pathogen-free conditions. WT cells and DDX58-KO cells (1 × 10^7^ cells) were suspended in 100 μl of PBS and subcutaneously injected into the right flank of 4-week-old female BALB/c nude mice. Tumour volume was calculated using the formula (V =0.52 × Length × Wide^2^). After almost 3 weeks, mice were euthanized when the average tumour volume reached 1000 mm^3^ (Cervical dislocation after Sodium pentobarbital injection). Tumours were collected and cut into almost 17 mm^3^ cubes and inoculated subcutaneously into nude mice. Fifteen days after injection of the tumour mass, the average tumour volume reached 200 mm^3,^ and drug therapy started. Dox was dissolved in PBS and given by intraperitoneal (i.p.) injection at 5 mg/kg every three days for 12 days. Control mice received the same amount of PBS by i.p. injection. Tumour volume was monitored during this time. On the 12th day after the initiation of therapy, all mice were euthanized. The tumour growth inhibition rate (IR) was calculated using the formula IR (%) = (1 – TWt/TWc) x 100, where TWt and TWc are the mean tumour weights of the treated and control groups, respectively.

### Immunofluorescence with anti-dsRNA J2 antibody

WT and DDX58-KO cells were seeded at 2x10^5^ cells per well in 12-well plates and treated with different concentrations of Dox. To serve as a positive control, poly(I:C) (10 μg/ml) (GLPBIO, GC14710) was added to the cells in the presence of Lipo3000. Six hours later, the cells were fixed with 100% methanol and blocked with 3% BSA in PBS (phosphate-buffered saline). A 1:100 dilution of anti-dsRNA antibody J2 monoclonal antibody (Nordic-MUbio,10010200) in 3% BSA in PBS was added into plates and incubated for 1 hr at room temperature (RT). Following three washes with 0.1% PBST (Triton-100 in PBS), the iFluor™ 488 anti-mouse antibody (HUABIO, HA1125) (1:100) was added to the plates and incubated for 1 hr at RT. Then, the plates were washed in 0.1% PBST three times, and diaminobenzidine tetrahydrochloride substrate (Sigma) was added for a 10-min incubation. Cells were visualized using an Imaging Universal microscope (Zeiss), and at least three fields per sample were counted.

### Western blotting

Protein was extracted using RIPA lysis buffer (Beyotime, P0013c) and quantified with the BCA Protein Assay Kit (Thermo Fisher Scientific, 23250). The extracted proteins were separated on a 10% polyacrylamide gel (Bio-Rad) and transferred to a PVDF membrane for immunoblotting. Blocking was performed in 5% low-fat milk in Tris-buffered saline containing 0.1% Tween (TBST) for 1 hr at RT. After blocking, the membranes were incubated in primary antibody dilutions overnight at 4°C. The membranes were incubated with mouse anti-rabbit (1:2000) and mouse anti-mouse (1:2000) (Abcam) for 1 hour at 37°C. Detection was performed using ECL Plus western blotting detection reagents (Millipore, USA).

### Cell counting kit-8 assay

Briefly, cells were seeded into 96-well plates at a density of 3000 cells per well. After incubation for the indicated time points, 10 μL of CCK-8 solution (MedChemExpress, HY-K0301) was added to 100 μL of cell medium in each well, followed by a 2 hrs incubation at 37°C with 5% CO2. The absorbance value (OD) was then measured at 450 nm using a microplate reader.

### Annexin V/7AAD and caspase 3/7 activity

After 24 hrs of Dox treatment, cells were harvested and stained with Annexin V-Alexa Fluor 647 (Biolegend, 640912) and 7AAD (Sigma–Aldrich, D9542) according to the manufacturer’s recommendation for apoptosis evaluation. Flow cytometry (CytoFLEX, C00445) was then used to analyse the stained samples, with each sample being tested in triplicate.

The Caspase 3/7 activity kit is a commercially available kit (KeyGEN BioTECH, KGAS037-100) used to measure the activity of caspase 3 and 7, which are key executioner caspases involved in apoptosis. The kit is designed to detect and quantify the activity of caspases 3 and 7 by measuring the enzymatic cleavage of a synthetic peptide substrate labelled with a fluorescent dye. The pictures were taken by Olympus microscope at low magnification (10x, objective), and at least three fields per sample were counted.

### Statistical analysis

Data analysis and mapping were performed using GraphPad Prism 9, and all data are presented as mean ± standard deviation (SD). Significance was determined by the unpaired Student’s-test.

## Results

### DDX58^low^ is associated with worse therapeutic efficacy and prognosis

**Figure 1:**
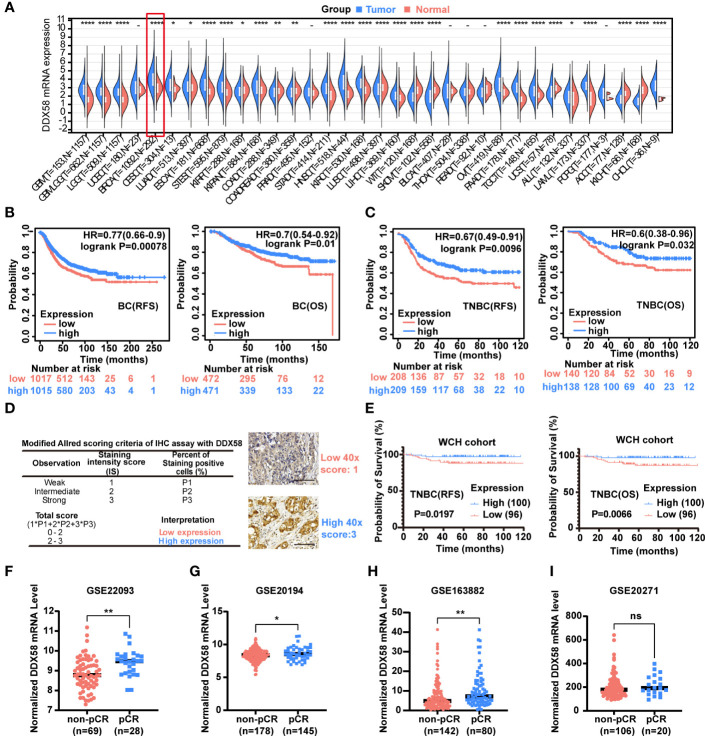
The *DDX58* gene is associated with the prognosis and chemotherapy efficacy of breast cancer. **(A)** Pan-cancer analysis of DDX58 gene using UCSC database. 34 types of cancer using R software (version 3.6.4) by unpaired Wilcoxon Rank Sum and Signed Rank Tests. T: tumour, N: normal (ns: no significance, *P < 0.05, **P < 0.01, ****P < 0.0001). **(B)** The prognosis (OS, RFS) of BC patients from the Kaplan–Meier plotter dataset. Blue: high expression, and Red: low expression. **(C)** The prognosis (OS, RFS) of TNBC patients from the Kaplan–Meier plotter dataset. Blue: high expression, and Red: low expression. **(D)** Modified Allred scoring system. Inserts show 40× magnifications, scale bar: 100 µm. **(E)** The prognosis (OS, RFS) of 196 TNBC patients among different expression level of *DDX58* expression. Blue: high expression, and Red: low expression. **(F-I)** The expression levels of DDX58 in patients with pCR and non-pCR from GSE22093, GSE20194, GSE163882 and GSE20271 datasets (ns: no significance, *P < 0.05, **P < 0.01) (Unpaired t test).

A Pan-cancer analysis was conducted to investigate *DDX58* differential expression between tumour and normal tissues by UCSC database (https://xenabrowser.net/). We found that *DDX58* expression was the highest expression level in breast cancer out of 34 types of cancer (**Figure 1A**). Of all the 34 cancer types, we also observed significant up-regulation of *DDX58* expression in 19 tumours, and down-regulation in 9 tumours compared to their normal tissues (**Figure 1A**).

To further explore the potential clinical implications of *DDX58* in breast cancer, we checked its prognosis significance in the Kaplan–Meier plotter dataset. Patients with low expression of *DDX58* (*DDX58* ^low^, using median as threshold) had significantly shorter recurrence-free survival (RFS) (*P*=0.00078) and overall survival (OS) (*P*=0.01) (**Figure 1B**). This phenomenon was more obvious in TNBC subgroup patients, who have significantly shorter RFS (*P*=0.0096) and OS (*P*=0.032) in *DDX58* ^low^ patients (**Figure 1C**). The prognosis of other BC subtypes was shown in **Supplementary Figure S1**. *DDX58* ^low^ patients only in Luminal A and HER2+ subtypes showed significantly shorter RFS (but no phenotype in OS) (**Supplementary Figure S1**). To confirm the essential role of *DDX58* in TNBC patients, we used a cohort of 196 female TNBC patients in our hospital, and semi-quantitative method for protein of immunohistochemistry (IHC) analysis was used. The baseline table of involved patients is shown in **Supplementary Table S1**. Based on the modified Allred scoring system (37, 38), we divided the patients into two groups: 100/196 (51%) patients with high *DDX58* expression (2≤score ≤ 3) and 96/196 (49%) patients with low *DDX58* expression (0≤score<2) (**Figure 1D**). The data of our hospital again confirmed that *DDX58* ^low^ patients in TNBC had worse prognosis for both RFS (*P*=0.0197) and OS (*P*=0.0066), which was consisting with the previous Kaplan–Meier plotter dataset results (**Figure 1E**).

To further investigate the effect of DDX58 on chemotherapy treatment efficacy in BC patients, we calculated the therapeutic effect of BC patients who underwent neoadjuvant therapy in four GEO datasets (**Figures 1F-I**). We found that the expression of *DDX58* was significantly higher in patients with a pathological complete response (pCR) in 3/4 of all datasheets (GSE20194, GSE22093, and GSE163882) (*P< 0.05, **P < 0.01) (**Figures 1F-H**). Taken together, our results indicated that the low expression of *DDX58* was significantly associated with worse therapeutic efficacy and poor clinical outcome. These data suggested that *DDX58* might be a potential novel predictive prognostic biomarker for BC.

### DDX58 knockdown leads to multiple chemo-drug resistance in TNBC

As *DDX58* ^low^ patients associated with worse therapeutic efficacy and poor clinical especially in TNBC patients, we hypothesized that DDX58 play crucial roles in the process of chemotherapy. Firstly, we silenced DDX58 with specific siRNA in TNBC (MDA-MB-231), luminal (MCF-7) and HER2-positive (SKBR3) breast cancer cells. Then, we conducted cell viability assays after treatment of a panel of commonly used chemotherapy agents for BC, including microtubule stabilizer (paclitaxel), DNA topoisomerase IIα (TOP2A) inhibitor (doxorubicin; Dox), antimetabolite (5-fluorouracil; 5-FU) and DNA alkylating agent [cis-diammineplatinum(II); cisplatin].

**Figure 2:**
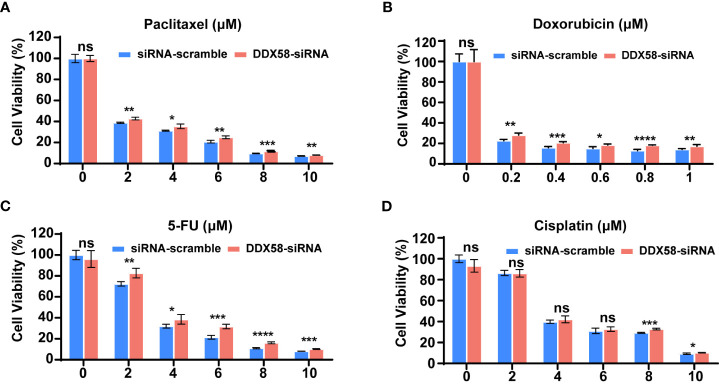
*DDX58* knockdown leads to chemotherapy drugs resistance in TNBC cells. **(A)** MDA-MB-231 cells were transiently transfected with DDX58-siRNA3 and siRNA-scramble. Cells were treated with increasing concentrations of paclitaxel for 24 hrs, then the proliferation rates of cells were measured by CCK8 assay. Student’s t test was used for comparisons (ns: no significance, *P < 0.05, **P < 0.01, ***P < 0.001). **(B)** MDA-MB-231 cells were transiently transfected with DDX58-siRNA3 and siRNA-scramble. Then cells were treated with increasing concentrations of Doxorubicin for 48 hrs and be measured by CCK8 assay (ns: no significance, *P < 0.05, ***P < 0.001, ****P < 0.0001). **(C)** MDA-MB-231 cells were transiently transfected with DDX58-siRNA3 and siRNA-scramble. Then cells were treated with increasing concentrations of 5-FU for 24 hrs and measured by CCK8 assay. (ns: no significance, *P < 0.05, **P < 0.01, ***P < 0.001, ****P < 0.0001). **(D)** MDA-MB-231 cells were transiently transfected with DDX58-siRNA3 and siRNA-scramble. Then cells were treated with increasing concentrations of Cisplatin for 24 hrs and be measured by CCK8 assay (ns: no significance, *P < 0.05, ***P < 0.001) (multiple t tests).

Interestingly, when *DDX58* expression was silenced in MDA-MB-231 cells, we observed significantly drug resistance after paclitaxel, Dox, or 5-FU treatment in *DDX58*-siRNA (DDX58-Knockdown) cells compared with the control cells (*DDX58*-scramble) (**Figures 2A-C**). This phenomenon was not observed in the group of cisplatin treatment (**Figure 2D**). Cisplatin bind to DNA and cause the DNA strands to crosslink, interfering the DNA replication process, subsequently inducing apoptosis, which is slightly different from the conventional paclitaxel, Dox, or 5-FU drugs (39, 40). However, for the other sub-types breast cancer cells, such as in MCF-7 cell and SKBR3, no dosage dependent manner chemo-drug resistance in *DDX58* gene knockdown group was observed, neither in paclitaxel, Dox, 5-FU nor in cisplatin treatment (**Supplementary Figure S2**).

In summary, all our data suggested that *DDX58* gene knockdown led to multiple line chemo-drug resistance in TNBCs rather than other BC subtypes. This phenomenon also explained why only TNBC patients with DDX58^low^ had worse treatment response and poor prognosis, while the *DDX58* ^low^ patients in other BC subtypes had no significant prognostic differences.

### DDX58 deficiency cause chemo-drug resistance in multiple TNBC cell lines

**Figure 3:**
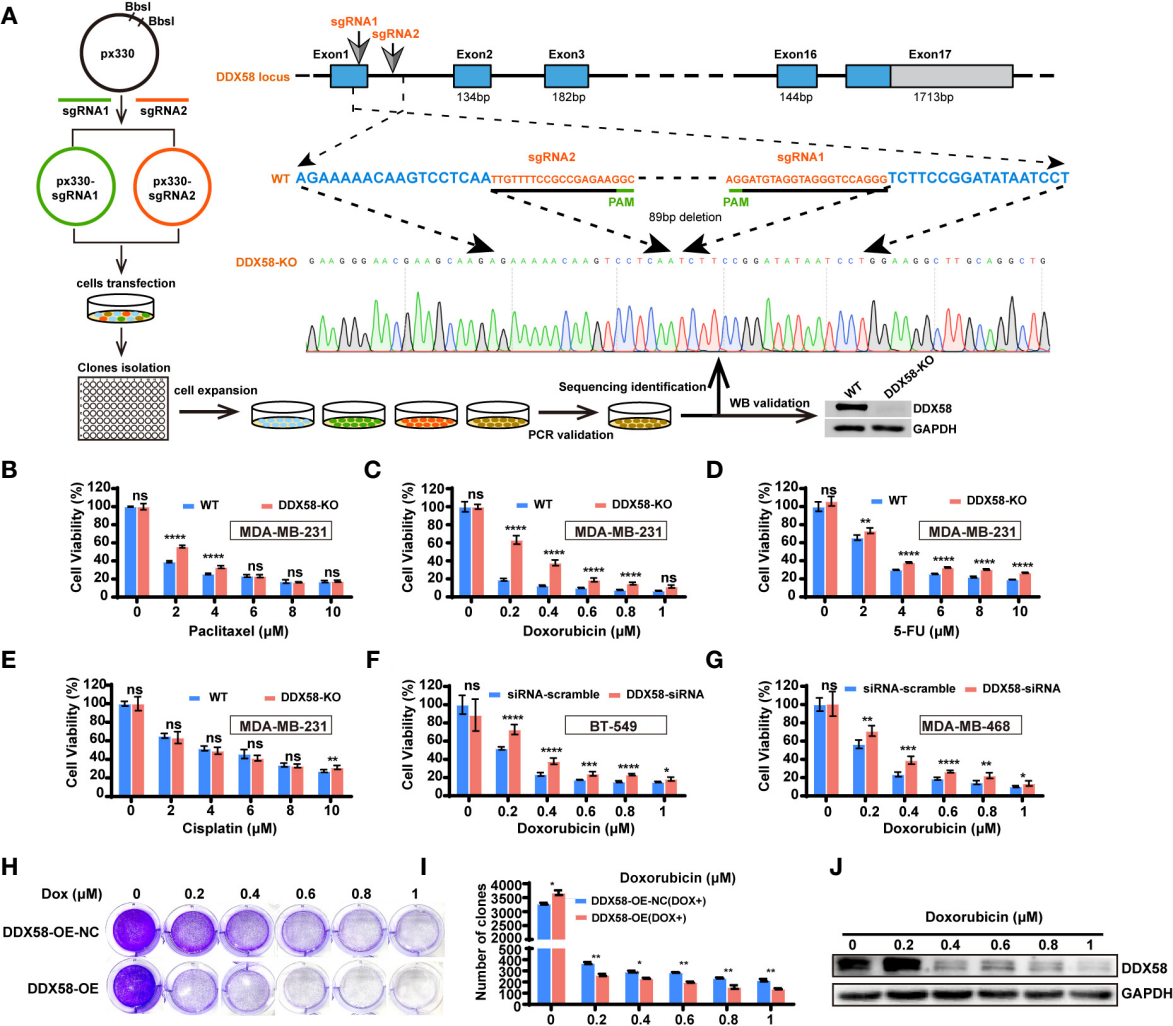
*DD*X58 deficiency was contributed to chemotherapy resistance in TNBC cells. **(A)** Schematic of DDX58 knockout cell line construction using the CRISPR/Cas9 system. **(B)** *DDX58*-KO cells were treated with increasing concentrations of paclitaxel and proliferation rates were measured by CCK8 assay (ns: no significance, ****P < 0.0001). **(C)** *DDX58*-KO cells were treated with Dox and proliferation rates were measured by CCK8 assay (ns: no significance, ****P < 0.0001). **(D, E)** *DDX58*-KO cells were treated with increasing concentrations of drugs (5-FU, cisplatin), and proliferation rates were measured by CCK8 assay (ns: no significance, **P < 0.01, ****P < 0.0001). **(F)** BT-549 cells were transiently transfected with *DDX58*-siRNA and siRNA-scramble. Cells proliferation rates were measured by CCK8 assay treated with increasing concentrations of Dox (ns: no significance, *P < 0.05, ***P < 0.001, ****P < 0.0001). **(G)** MDA-MB-468 cells were transiently transfected with *DDX58*-siRNA and siRNA-scramble. Cells proliferation rates were measured by CCK8 assay after treated with increasing concentrations of Dox (ns: no significance, *P < 0.05, **P < 0.01, ***P < 0.001, ****P < 0.0001). **(H)** The *DDX58*-OE and control cells (DDX58-OE-NC) were treated with different concentrations of Dox and stained with crystal violet after Dox treatment. **(I)** The statistical results of the crystal violet staining (*P < 0.05, **P < 0.01) (multiple t tests). **(J)** Western blotting was used to detect the expression of DDX58 protein under a gradient concentration of Dox in *DDX58*-OE cells.

To further confirming the chemo-drug resistance effect in DDX58^low^ in TNBC cells, we first knocked out *DDX58* gene in TNBC cells (MDA-MB-231) for the chemo-drug effect testing, and then we confirmed this in other TNBC cell lines by silencing *DDX58*. We knocked out this gene by the CRISPR/Cas9 system in MDA-MB-231 cells (*DDX58*-KO) (**Figure 3A**). Sanger sequencing and western blotting were used to confirm the knockout efficiency of this gene in DNA and protein level (**Figure 3A**). *DDX58*-KO and wildtype cells (WT) were treated with four types chemotherapy drugs mentioned above, and cell viability assays were carried out to detect drug efficiency. Our results demonstrated that *DDX58*-KO cells showed significant drug resistance under the treatment of three chemotherapy drugs including paclitaxel (**Figure 3B**), Dox (**Figure 3C**) and 5-FU (**Figure 3D**), except for cisplatin (**Figure 3E**). Cells were found to be the most resistant to Dox in strictly dose-dependent manner among all these drugs (**Figure 3C**). To verify this phenomenon in other TNBC cell lines, we silenced *DDX58* gene by siRNA in BT-549 and MDA-MB-468 to confirm these results under treatment of Dox. Interestingly, Dox resistance was also observed in *DDX58* gene silenced groups compared with in the WT group (**Figures 3F, G**). Thus, these results demonstrated that *DDX58* gene deficiency caused chemo-drug resistance in multiple TNBC cell lines.

To reversely demonstrate the essential role of DDX58 gene in the process of chemo-drug treatment, we constructed a DDX58 stably overexpression cell line (DDX58-OE) and the control cell line (DDX58-OE-NC) in MDA-MB-231 cells. After four chemotherapy drug treatments, we observed that DDX58-OE cells were more sensitive to drugs than DDX58-OE-NC cells, especially in Dox treatment (**Supplementary Figure S3**). Significantly fewer cells survived in the DDX58-OE group than the DDX58-OE-NC group within 48 hrs treatment of Dox (**Figure 3H**), and this phenomenon was also in a dose-dependent manner (**Figure 3I**). Very interestingly, survived cells tended to express fewer DDX58 protein level after a series of gradient Dox concentration treatment (**Figure 3J**). This data suggests that TNBC cells with high DDX58 expression are more sensitive to Dox treatment. In summary, using *DDX58*-KO/*DDX58*-KD and *DDX58*-OE in multiple TNBC cells, we demonstrated that cells with low DDX58 expression were resistant to multiple chemotherapy, e.g. paclitaxel, Dox, 5-FU, while cells with high DDX58 expression were sensitive to Dox treatment.

### DDX58 deficiency decrease cell proliferation but increases cell invasion and migration *in vitro*

**Figure 4:**
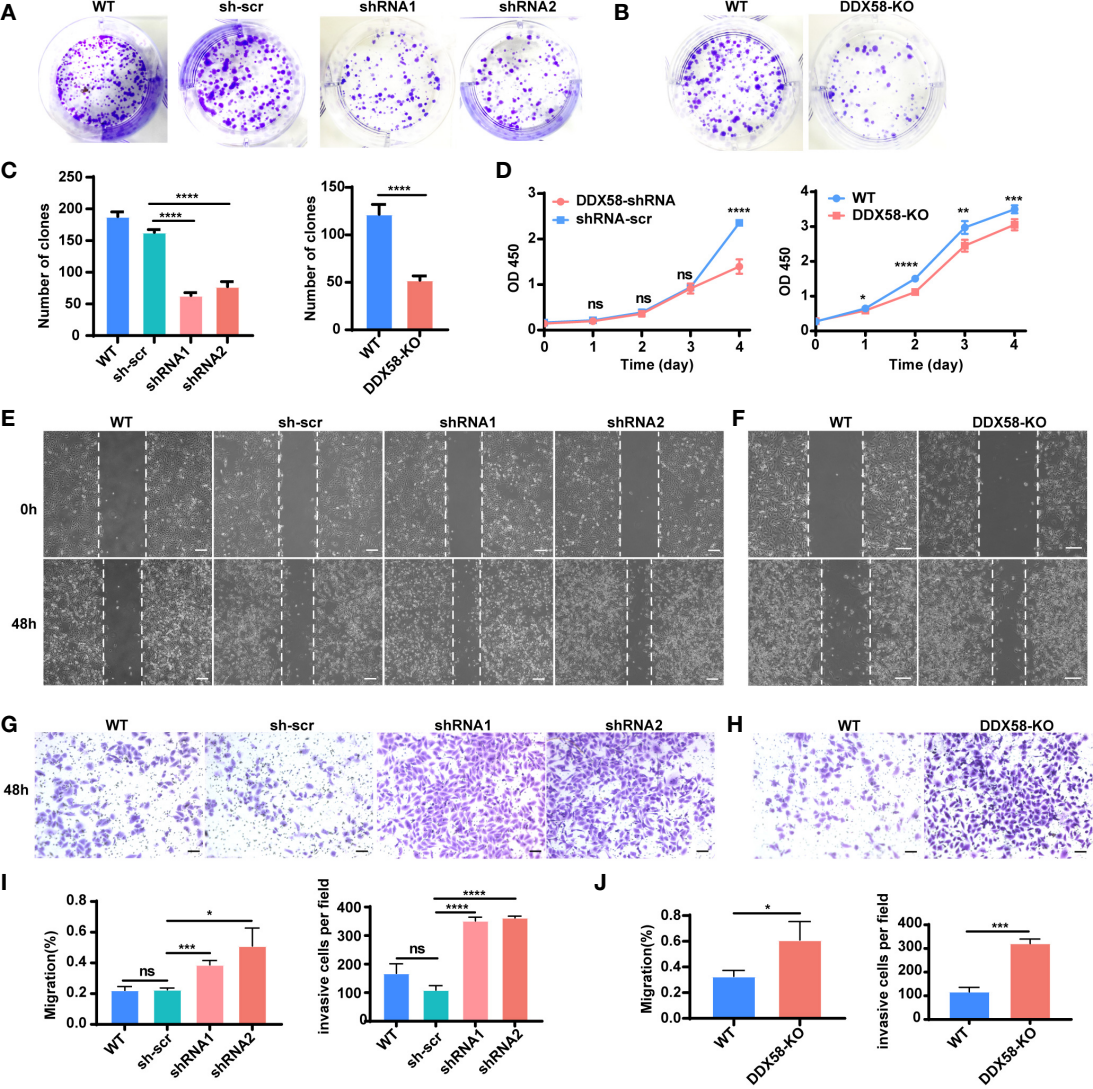
Depletion of DDX58 inhibited proliferation but induced migration and invasion in TNBC cells. **(A)** Colony formation of DDX58-shRNA cells after 7 days. **(B)** Colony formation of DDX58-KO cells after 7 days. **(C)** The statistical results of clones in every experimental group (****P < 0.0001). **(D)** CCK8 assay results showing that the effect of DDX58 expression on cell growth in MDA-MB-231 cells (ns: no significance, *P < 0.05, **P < 0.01, ****P < 0.0001, ***P < 0.001). **(E)** Wound healing assay was used to detect the migration of DDX58-shRNA cells. Photographs were taken at 0 and 48 hrs following the initial scratch. The size of the scale bar is 200 µm. **(F)** Wound healing assay was used to detect the migration of DDX58-KO cells. Photographs were taken at 0 and 48 hrs following the initial scratch. The size of the scale bar is 200 µm. **(G)** Cell invasion assays were performed in DDX58-shRNA cells by using Matrigel-coated transwell chambers. The size of the scale bar is 100 µm. **(H)** Cell invasion assays were performed in DDX58-KO cells by using Matrigel-coated transwell chambers. The size of the scale bar is 100 µm. **(I)** Migration rates were quantified by measuring wound areas. Image J software was used in measuring (ns: no significance, *P < 0.05, ****P < 0.0001, ***P < 0.001). **(J)** The statistical results of invasion cells of each experimental group (*P < 0.05, ***P < 0.001) (Unpaired t test).

To further investigate the underlying mechanisms by which *DDX58* affects chemotherapy resistance and prognosis in breast cancer, we conducted a functional exploration of *DDX58* on the typical biological characteristics of cancer cells. Specifically, we constructed *DDX58* gene knockdown (DDX58-shRNA) MDA-MB-231 cell lines. Firstly, we found that the number of monoclonal formations was inhibited in *DDX58*-shRNA compared to the control group (**Figure 4A**), and the similar results were further confirmed in *DDX58*-KO cells (**Figure 4B**), indicating that *DDX58*-shRNA and *DDX58*-KO led to impaired cancer cell growth and proliferation. The number of clones were calculated in every experimental group and the results was shown in **Figure 4C**. Secondly, our results indicated that the viabilities of *DDX58*-shRNA cells were significantly decreased, also the same results were found in *DDX58*-KO cells (**Figure 4D**). These above results further supporting the importance of *DDX58* in maintaining cancer cell survival and proliferation.

Furthermore, we found that the migration of DDX58-shRNA cells enhanced (**Figure 4E**), and this phenomenon was further confirmed in DDX58-KO cells (**Figure 4F**). Then, we observed an enhancement in the invasion of DDX58-shRNA and DDX58-KO cells (**Figures 4G, H**). The statistical results of each experimental group were showed in **Figures 4I, J**. These results demonstrated that the migration and invasion of *DDX58*-deficient cells were enhanced. In conclusion, our data indicated that the depletion of *DDX58* inhibited proliferation and promoted the migration and invasion in MDA-MB-231 cells.

### dsRNA-DDX58-MAVS signalling pathway is activated by chemo-drug induced double-stranded RNAs

**Figure 5:**
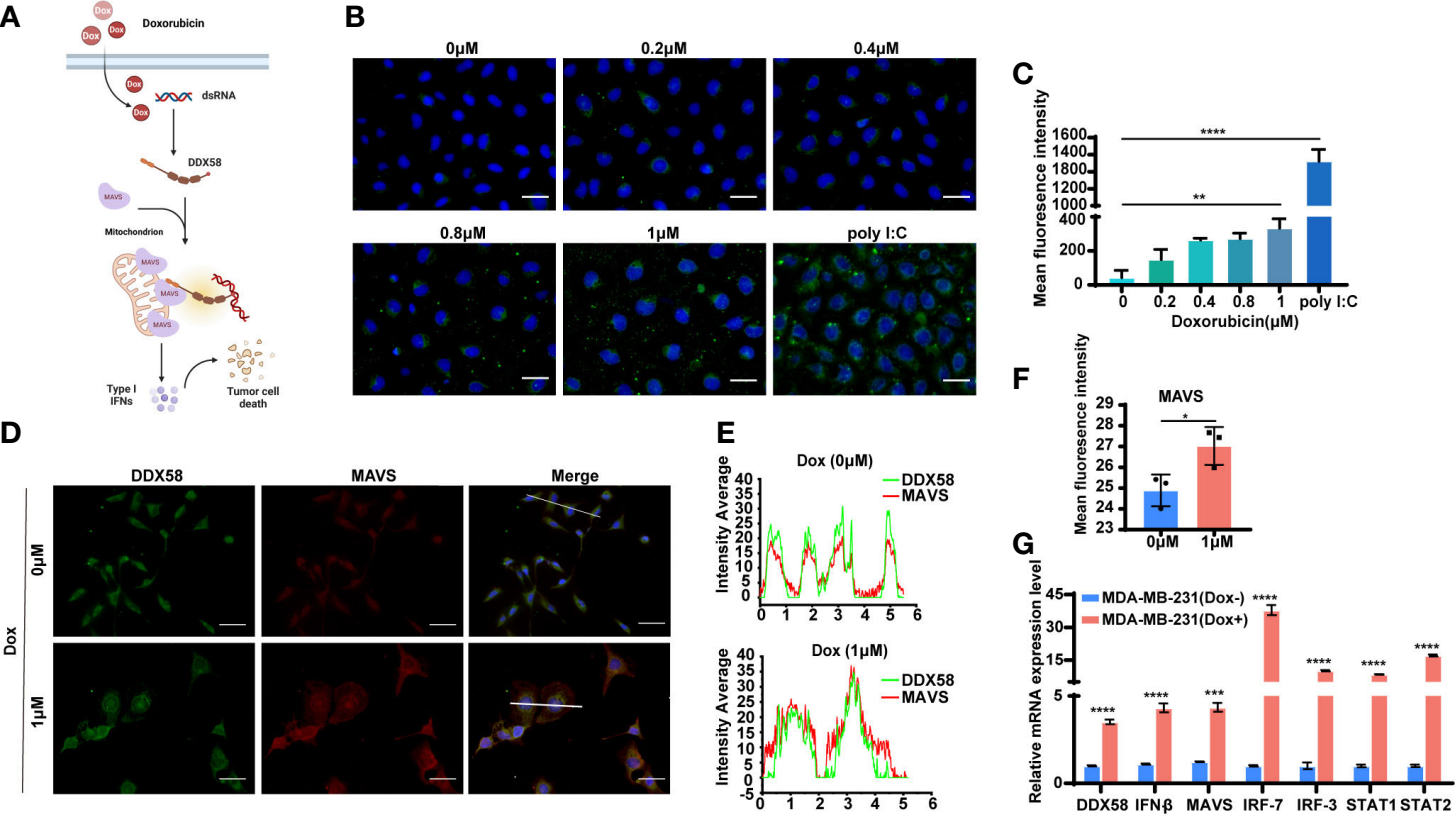
The DDX58 and downstream signalling pathway was activated by Dox-induced endogenous double-stranded RNAs. **(A)** Proposed mechanism of DDX58 dependent activation of Type I IFN signalling in the cellular response to Dox. **(B)** Immunofluorescence staining of dsRNA in MDA-MB-231 cells with anti-dsRNA J2 antibody (green) and DAPI (blue). Cells transfected with poly I:C were included as a positive control for dsRNAs. The size of the scale bar is 100 µm. **(C)** Data statistics of immunofluorescence of dsRNA J2 were performed in average grey values by Image J software (**P < 0.01, ****P < 0.0001). **(D)** Double fluorescence staining of MAVS and DDX58 in DDX58-OE cells with anti-DDX58 antibody (green), anti-MAVS antibody (red) and DAPI (blue). The size of the scale bar is 100 µm. **(E)** The analysis of immunofluorescence colocalization was performed by Origin 2018 software. **(F)** Data statistics of IF of MAVS were performed in average grey values by Image J software (*P < 0.05). **(G)** RT-qPCR was used to confirm the expression DDX58 and downstream Type I IFN signalling pathways (***P < 0.001, ****P < 0.0001) (multiple t tests).

DDX58, an RNA sensor, is maintained in an inactive conformation without recognizing dsRNAs (double-stranded RNA) (41). Upon encounter with the cytoplasmic dsRNAs, the receptor of DDX58 changes conformation and releases a pair of signalling domains (CARDs) that are selectively modified and interact with an adapter protein (MAVS), thereby leading to induction of Type I IFN, proinflammatory cytokines and apoptosis (41, 42). According to this, we drew the hypothesis diagram of associate signal pathway with Dox treatment in TNBC cells (**Figure 5A**). First, we checked the endogenous expression level of dsRNAs in MDA-MB-231 cells following 6 hrs Dox treatment. Poly(I:C) (10 μg/ml) was used as positive control. After 6 hrs Dox treatment, the results of immunofluorescence (IF) staining of dsRNAs revealed a significant dose-dependent increasing (8-fold) in MDA-MB-231 cells (Dox concentration: 1 µM) (**Figures 5B, C**). To further investigated the downstream signalling pathway of DDX58 involved after 6 hrs Dox treatment, we performed double fluorescence staining of MAVS and DDX58. The results revealed that the expression of MAVS was significantly induced (**Figures 5D, F**) and two proteins (DDX58, MAVS) showed the increased co-localization after Dox treatment (**Figures 5D**, **4E**). Moreover, the data of RT-qPCR indicated that the expression of DDX58 and downstream Type I IFN signalling pathway genes significantly increased under 6 hrs Dox treatment, suggesting that Dox treatment could activate the DDX58-Type I IFN signalling pathway in MDA-MB-231 cells (**Figure 5G**). Taken together, our results confirms that Dox treatment elevates the level of endogenous dsRNAs, which then activates DDX58. The activated DDX58 interact with MAVS and the subsequent Type I IFN signalling pathway were induced.

### *DDX58*-KO interrupted chemo-drug induced cell apoptosis

**Figure 6:**
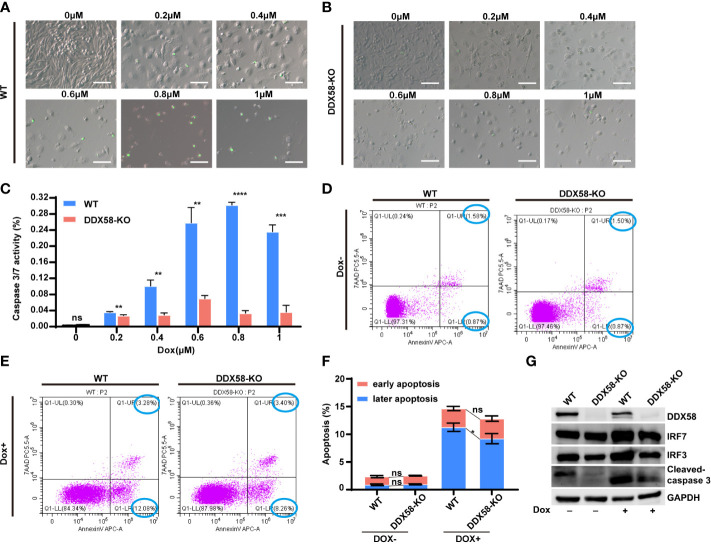
The depletion of *DDX58* mediates inhibited drug-induced cell apoptosis. **(A)** After 48 hrs Dox treatment, Caspase 3/7 activity of WT cells was determined using the Caspase 3/7 Activity Kit according to the manufacturer’s instructions. The size of the scale bar is 100 µm. **(B)** After treatment with different Dox concentrations, Caspase 3/7 activity of DDX58-KO cells was determined using the Caspase 3/7 Activity Kit. **(C)** The statistical results of caspase 3/7 activity (**P < 0.01, ***P < 0.001, ****P < 0.0001). **(D)** After Dox (1μM) treatment for 48h, cells were analysed by flow cytometry after staining with Annexin V-Alexa Fluor 647 and 7AAD according to the manufacturer’s recommendations. **(E)** After Dox (1μM) treatment for 48 hrs, DDX58-KO cells were analysed by flow cytometry. **(F)** The statistical results of flow cytometry data (*P < 0.05). **(G)** The expression of apoptosis and Type I IFN related genes were detected by western blotting after Dox (1μM) treatment. GAPDH was used as an internal control.

Previous studies have reported the activation of DDX58 and Type I IFNs signalling, which cause damaged cells apoptosis (10, 12–16, 43–45). Our above data also confirmed that the *DDX58* signalling and downstream Type I IFN signalling could be activated by Dox treatment in TNBC cells. These evidences indicated that the deficiency of *DDX58* might decrease cell apoptosis induced by Dox treatment, and finally leading to chemotherapy resistance. To further explore specific molecular event, we performed apoptosis assays in *DDX58*-KO and WT cells. Specifically, we utilized a caspase 3/7 kit and flow cytometry measurements for apoptosis testing. Detection of caspase 3/7 is a commonly used method for assessing apoptotic activity in experimental cell populations. Our data indicated that caspase 3/7 activity in *DDX58*-KO cells did not change significantly with an increasing Dox dosage concentration, however, the activities of WT cells significantly increased in a Dox treatment dose-dependent manner (**Figures 6A-C**). Moreover, without Dox treatment, the results of flow cytometry revealed that there was no significant difference in early and later apoptosis between *DDX58*-KO and WT cells (**Figures 6D, F**). Under Dox treatment, the later phase apoptosis rate of DDX58-KO cells was significantly lower than that of WT cells (**Figures 6E, F**).

IRF3/7 (interferon regulatory factor 3/7) is transcriptionally induced in many cell types as a target of Type I IFN signalling pathway (9, 46, 47). Our western blotting data showed that the expression of IRF3/7 did not change significantly without Dox treatment (**Figure 6G**). Nevertheless, under the treatment of Dox, the expression of IRF3/7 and cleaved caspase 3 increased in WT cells, comparing to *DDX58*-KO cells (**Figure 6G**). These results further indicated that DDX58-Type I IFN signalling was activated by Dox. Apoptosis is also commonly reported to be the downstream of DDX58-Type I IFN signalling pathway. Therefore, our data indicated that knockout of *DDX58* ultimately interrupted Dox-induced apoptosis, which was previously confirmed activated by DDX58-Type I IFN signalling pathway.

### *DDX58-*KO caused chemotherapy resistance by interrupting chemo-drug induced apoptosis *in vivo*

**Figure 7:**
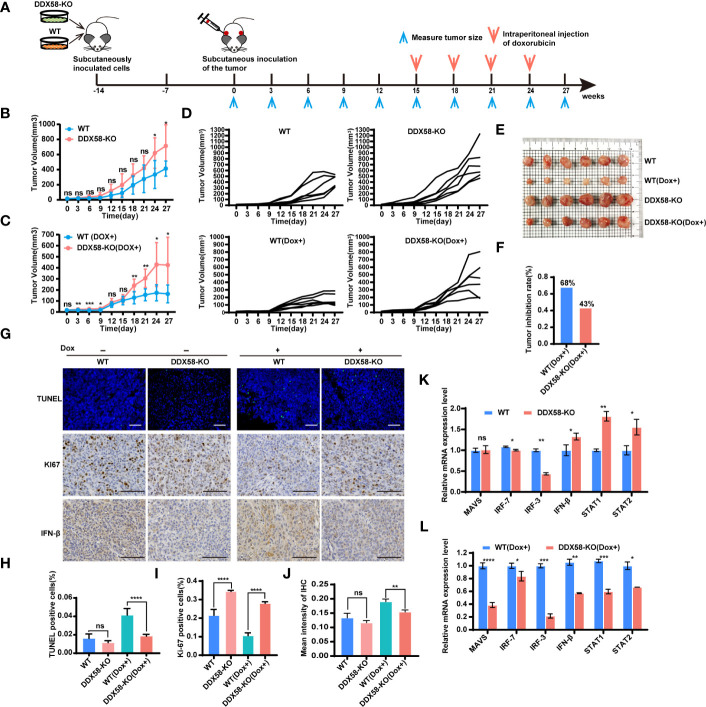
*DDX58*-deficient cells induced Dox resistance and inhibited DDX58-associated signalling pathways in CDX models. **(A)** Schematic of CDX models and dox treatment regimen. **(B)** Tumour growth curve of mice from the DDX58-KO group and the WT group (ns: no significance, *P < 0.05). **(C)** Tumour growth curve of mice from the DDX58-KO group and the WT group with Dox treatment (ns: no significance, *P < 0.05, **P < 0.01, ***P< 0.001) **(D)** The tumour growth curve of each nude mouse from four groups (DDX58-KO, WT, DDX58-KO (Dox+), WT(Dox+)). **(E)** After 27 days of tumour growth, including 12 days of Dox treatment, tumours in mice were excised and photographed for each group. **(F)** The tumour growth inhibition rates of the two groups (DDX58-KO, WT) were compared in the histogram. **(G)** TUNEL/KI67/IFN-β staining of tumour slices from each group. Apoptotic cells were stained by TUNEL (green). The scale bar is 100 μm and applied for all images. **(H)** The numbers of TUNEL-positive cells were calculated and compared. Data are expressed as the mean ± SE. no significance(ns) and ****P < 0.0001. **(I)** The numbers of Ki67-positive cells were calculated and compared (****P < 0.0001). **(J)** Statistical analysis of immunohistochemistry staining intensity using ImageJ (ns: no significance, **P < 0.01). **(K, L)** RT-qPCR was used to detected the expression of marker genes from *DDX58* and downstream Type I IFN signalling pathways in tumours of *DDX58*-KO and WT group (ns: no significance, *P < 0.05, **P < 0.01, ***P < 0.001, ****P < 0.0001) (multiple t tests).

To further reveal the role of *DDX58* in the process of Dox treatment *in vivo*. We established a cell line-derived xenograft (CDX) model in BALB/c nude mice using *DDX58*-KO and WT cells. The experimental design and Dox therapy regimen are depicted in **Figure 7A**. On days 24 and 27 post tumour implantation, the tumour volumes in the *DDX58*-KO group were significantly increased compared to those in the WT group (*P*< 0.05) (**Figure 7B**). This data indicates that *DDX58* knockout can promote tumorigenesis *in vivo*. We next evaluated the impact of *DDX58* in the process of Dox treatment based on these CDX model. After 12 days Dox treatment, the tumour volume of the DDX58-KO group was significantly greater than that of the WT group, with a 2.5-fold increasing (**Figure 7C**). The tumour growth curves of each nude mouse from the four groups (DDX58-KO, WT, DDX58-KO (Dox+), and WT (Dox+)) were shown in **Figure 7D**. Following 12 days of Dox treatment, tumours were harvested and photographed (**Figure 7E**). From these tumours, we found that the tumour growth inhibition rate (IR) of the *DDX58*-KO group (43%) decreased by 25% compared with that of the WT group (68%) (**Figure 7F**). These data, consistent with the results *in vitro*, revealed that knockout of *DDX58* leads to Dox chemotherapy resistance *in vivo.*

To further investigate the molecular event of Dox resistance associated with *DDX58 in vivo*, we detected apoptosis-related markers in tumours (**Figure 7G**). The IF results demonstrated that there was a marked reduction in the percentage of TUNEL-positive cells in the *DDX58*-KO group under Dox treatment (**Figures 7G, H**). Conversely, the percentage of Ki67-positive cells in the *DDX58*-KO group increased significantly compared to that in the WT group within Dox dosing (**Figures 7G, I**). Furthermore, the expression of interferon-beta (IFN-β) (a marker of the Type I IFN signalling pathway) decreased significantly in the tumours of the DDX58-KO group compared to the WT group following Dox treatment (**Figures 7G, J**). We further used RT–qPCR to check the expression of marker genes of DDX58*-*Type I IFN signalling pathways with Dox treatment *in vivo* (**Figures 7K, L**). Among the groups without Dox treatment, we found that marker genes from DDX58-Type I IFN signalling pathway had no significant differences in *DDX58*-KO group compared to the WT group. However, under the treatment of Dox, the expression of all markers significantly decreased in the *DDX58*-KO group compared to WT group (**Figure 7L**). These results further confirmed that the DDX58 signalling pathway and downstream Type I IFN signalling pathway could be activated by Dox treatment *in vivo*.

Taken together, our findings provide evidence that *DDX58* knockout promotes tumorigenesis and impedes the therapeutic efficacy of Dox in TNBC. The observed effects of *DDX58* knockout highlight the potential role of *DDX58* as a therapeutic target for TNBC treatment.

## Discussion

Due to TNBC’s aggressive biological properties and lack of molecular therapeutic targets, its poor overall survival has remained a severe clinical issue over the past decades. Although most TNBC cases initially respond well to chemotherapy, a substantial proportion of patients eventually relapse due to drug resistance (32). Identifying corresponding biomarkers and potential therapeutic targets for chemotherapy resistance is beneficial in improving the prognosis for TNBC patients. In this study, we investigated the role of the *DDX58* gene in TNBC prognosis and its contribution to chemo-drugs treatment. Our experimental results demonstrated that *DDX58* had a strong correlation with TNBC prognosis, and the deficiency of DDX58 led to Dox chemotherapy resistance by inhibiting cell apoptosis, which normally should be induced by the DDX58-Type I IFN signalling pathway in Dox treatment.

The development of chemoresistance in TNBC is a complex process driven by multiple signalling pathways. It is well known that hypoxia signalling pathway has a clear connection with chemoresistance (48). NF-κB pathway activation mediates chemotherapy resistance in breast cancer and other types of cancer (49). Preclinical data demonstrated that Notch signalling is crucial for TNBC chemoresistance, and provided the evidence that Notch inhibitors could sensitize TNBC cells to cytotoxic agents (50, 51). Additionally, Other associated signalling pathways, such as the Wnt (52), PTEN (53) and Hedgehog (54) signalling pathways, have been reported to be associated with chemoresistance. Nevertheless, none of these mechanisms have been effectively applied clinically. In addition, it is well known that evading apoptosis is a major hallmark of cancer. And some apoptosis-related signalling pathways are linked with resistance to various cytotoxic agents, which are critical for TNBC treatment (55, 56). Consequently, targeting dysregulated apoptosis and increasing the susceptibilities of tumour cells to chemotherapy drugs could improve the cancer chemotherapy response (55, 56). TNBC patients might appear to benefit greatly from the development of new therapeutics targeting apoptosis-associated pathways. However, there is still a shortage of specific biomarkers of resistance associated with apoptosis and underlying mechanisms of chemotherapy drug resistance. Based on this, our study discovered that the apoptosis effect of chemotherapy drugs is decreased by the low expression of DDX58, offering specific drug resistance biomarkers and a potential therapeutic strategy for chemotherapy in TNBC.

Previous studies have shown that *DDX58* is a proto-oncogene, an important immune-related gene closely related to immune cell infiltration. Its high expression promotes the carcinogenesis of cancers (57). Similarly, our results indicated that the expression of *DDX58* in tumour samples was significantly higher than that in normal samples of TNBC (**Figure 1A**). And the deficiency of DDX58 inhibited proliferation but induced migration and invasion in TNBC cells (**Figure 4**). However, in animal experiments, we found that tumours in the DDX58-KO group grew faster than those in the control group (**Figure 7B**). The apparent discordance between cell and animal models could be attributed to several factors. Firstly, cells and animals grow in diverse environments, and variations in environmental factors may lead to divergent results. Secondly, gene deletion may activate compensatory mechanisms, which may exhibit disparate behaviours in different models. Lastly, gene deletion may perturb the expression and function of other genes, which may have dissimilar effects in distinct models. Thus, further comprehensive investigations and explorations are warranted to elucidate the underlying reasons for the divergent effects of DDX58 gene deletion in cell and animal models.

In this study, we first investigated the expression and clinical implications of *DDX58* in breast cancer. Our data demonstrated that patients with low expression of DDX58 had worse treatment response and poor prognosis in TNBC rather than other BC subtypes. Then this phenomenon might be explained by that DDX58-knockdown led to multiple line chemo-drug resistance in TNBC rather than other BC subtypes. *DDX58*, a conserved member in the family of dsRNA-binding proteins, which also includes the innate immune surveillance proteins MDA5 and LGP2 (41), serves as a cytoplasmic pattern recognition receptor that detects viral RNAs during infection, thereby initiating antiviral signalling pathways. This activation leads to the production of Type I interferons (IFNs) and other proinflammatory cytokines (58, 59). Our experiments demonstrates that Dox treatment enriched the endogenous dsRNAs (**Figures 5B, C**) and upregulated the expression of MAVS (**Figures 5D, F**). Subsequent activation of MAVS initiated downstream signalling pathways, including the Type-I IFN signalling pathway (**Figure 5G**), which exerts direct anticancer effects by activating apoptosis (60, 61).

IFNs are cytokines that play a central role in initiating immune responses, especially antiviral and antitumour effects (62). There are three types of IFNs: Type I (IFN-alpha, IFN-beta, and others, such as omega, epsilon, and kappa), Type II (IFN-gamma), and Type III (IFN-lambda) (62). Notably, increasing evidence highlights the importance of IFN signalling pathways in the response to ionizing radiation (IR) and cancer therapy. The IFN signalling pathway is activated by radiotherapy, and then multiple interferon stimulator genes (ISGs) are activated with simultaneous growth arrest and death of cancer cells (63–66). New evidence has demonstrated that IR and chemotherapy activate Type I IFN signalling in tumour and host cells, and IR stimulates the binding of cytoplasmic DDX58 to small endogenous noncoding RNAs (sncRNAs), thereby activating the Type I IFN signalling pathway (27). Nevertheless, the IFN signalling pathway has been relatively less reported in cancer chemotherapy. Remarkably, our findings revealed that DDX58 deficiency could inhibit cell apoptosis by DDX58 and the downstream Type I IFN signalling pathways during Dox treatment (**Figure 6**). These results provide new insights into the relationship between the DDX58 signalling pathway and the chemotherapy resistance of TNBC cells.

Elucidating the mechanisms of chemotherapy resistance helps identifying therapeutic targets for precision medicine, ultimately improving cancer survival, e.g. for TNBC, which lacks effective therapeutic targets. Our experimental results showed that *DDX58* contributes to Dox chemotherapy by inducing Type I IFN signalling-induced apoptosis in TNBC cells. Furthermore, our *in vivo* study demonstrated the potential therapeutic benefit of *DDX58* expression in combination with Dox treatment. However, several unresolved scientific questions remain, including the mechanisms of dsRNAs enrichment after Dox treatment in TNBC cells and the possibility of reversing Dox resistance by overexpressing DDX58 in DDX58-KO cells. Whether other RLR family members, including MDA5 and LGP2, are also involved in Dox-related signalling pathways still needs further investigation. In summary, further studies are needed to fully elucidate the clinical significance of *DDX58* in TNBC and to optimize its usage as a predictive biomarker as well as a full assessment for therapeutic targets.

## Conclusions

In summary, our study provides mechanistic insights into the role of *DDX58* in Dox chemotherapy treatment of TNBC and the mechanism for chemotherapy resistance in DDX58^low^ TNBC patients. The enrichment of endogenous dsRNAs by Dox treatment in TNBC cells triggers the activation of MAVS, which leads to the downstream activation of the Type I IFN signalling pathway and subsequent apoptosis. Importantly, we demonstrate that *DDX58* knockout inhibits this pathway and confers resistance to Dox treatment. These findings highlight the therapeutic potential of targeting *DDX58* to sensitize TNBC cells to Dox and suggest a strategy for individualizing treatment regimens based on DDX58 expression status.

## Data availability statement

The original contributions presented in the study are included in the article/**Supplementary Material**. Further inquiries can be directed to the corresponding author.

## Ethics statement

The study was approved by the Clinical Test and Biomedical Ethics Committee of West China Hospital Sichuan University (2022-1056). The studies were conducted in accordance with the local legislation and institutional requirements. The human samples used in this study were acquired from primarily isolated as part of your previous study for which ethical approval was obtained. Written informed consent for participation was not required from the participants or the participants’ legal guardians/next of kin in accordance with the national legislation and institutional requirements.

## Author contributions

SC: Conceptualization, Data curation, Formal analysis, Investigation, Methodology, Project administration, Software, Supervision, Validation, Visualization, Writing – original draft, Writing – review & editing. XL: Data curation, Formal analysis, Investigation, Methodology, Software, Validation, Writing – review & editing. LX: Data curation, Investigation, Methodology, Software, Writing – review & editing. PZ: Formal analysis, Investigation, Methodology, Validation, Writing – review & editing. MS: Formal analysis, Methodology, Software, Writing – original draft. FC: Formal analysis, Methodology, Software, Writing – review & editing. CB: Data curation, Formal analysis, Software, Writing – review & editing. XZ: Conceptualization, Funding acquisition, Resources, Supervision, Visualization, Writing – review & editing. TL: Funding acquisition, Resources, Supervision, Validation, Visualization, Writing – review & editing. FY: Conceptualization, Funding acquisition, Project administration, Resources, Supervision, Validation, Visualization, Writing – original draft, Writing – review & editing.
