# Supplementary figures and images for: DDX58 deficiency leads to triple negative breast cancer chemotherapy resistance by inhibiting Type I IFN-mediated signalling apoptosis
Source: Front Oncol. 2024 Mar 14;14:1356778. doi: 10.3389/fonc.2024.1356778 (PMC10974639; doi:10.3389/fonc.2024.1356778)

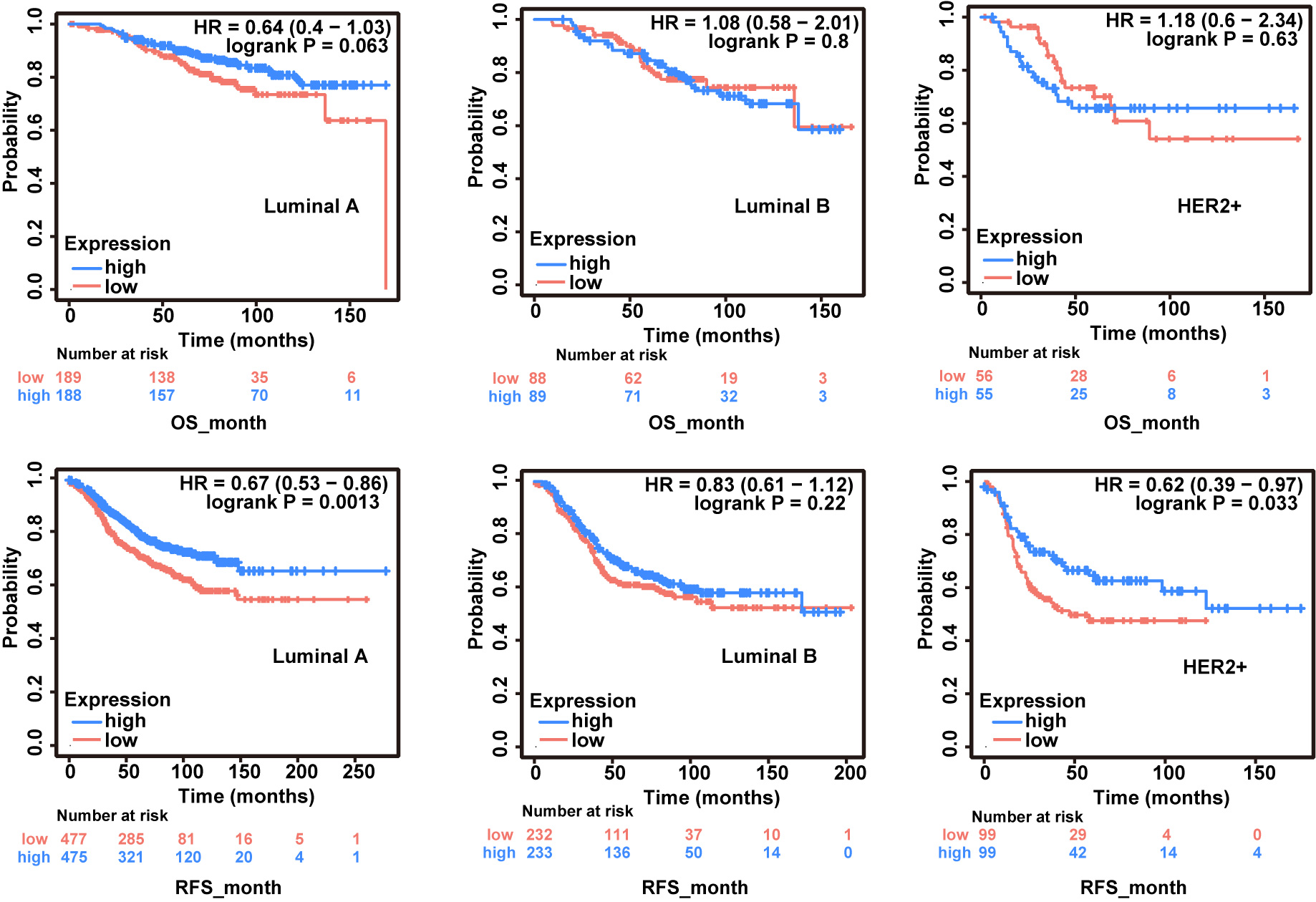

Supplement: Supplementary file 1 [file Image_1.jpg]

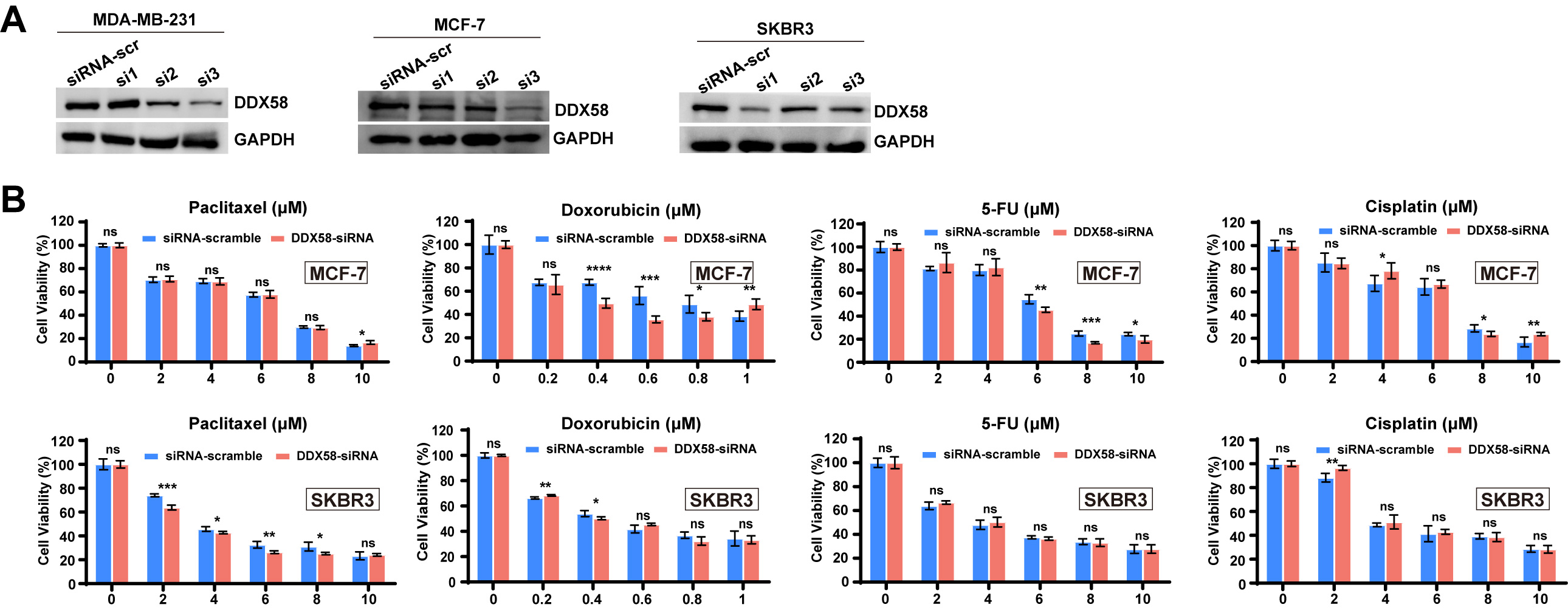

Supplement: Supplementary file 2 [file Image_2.jpg]

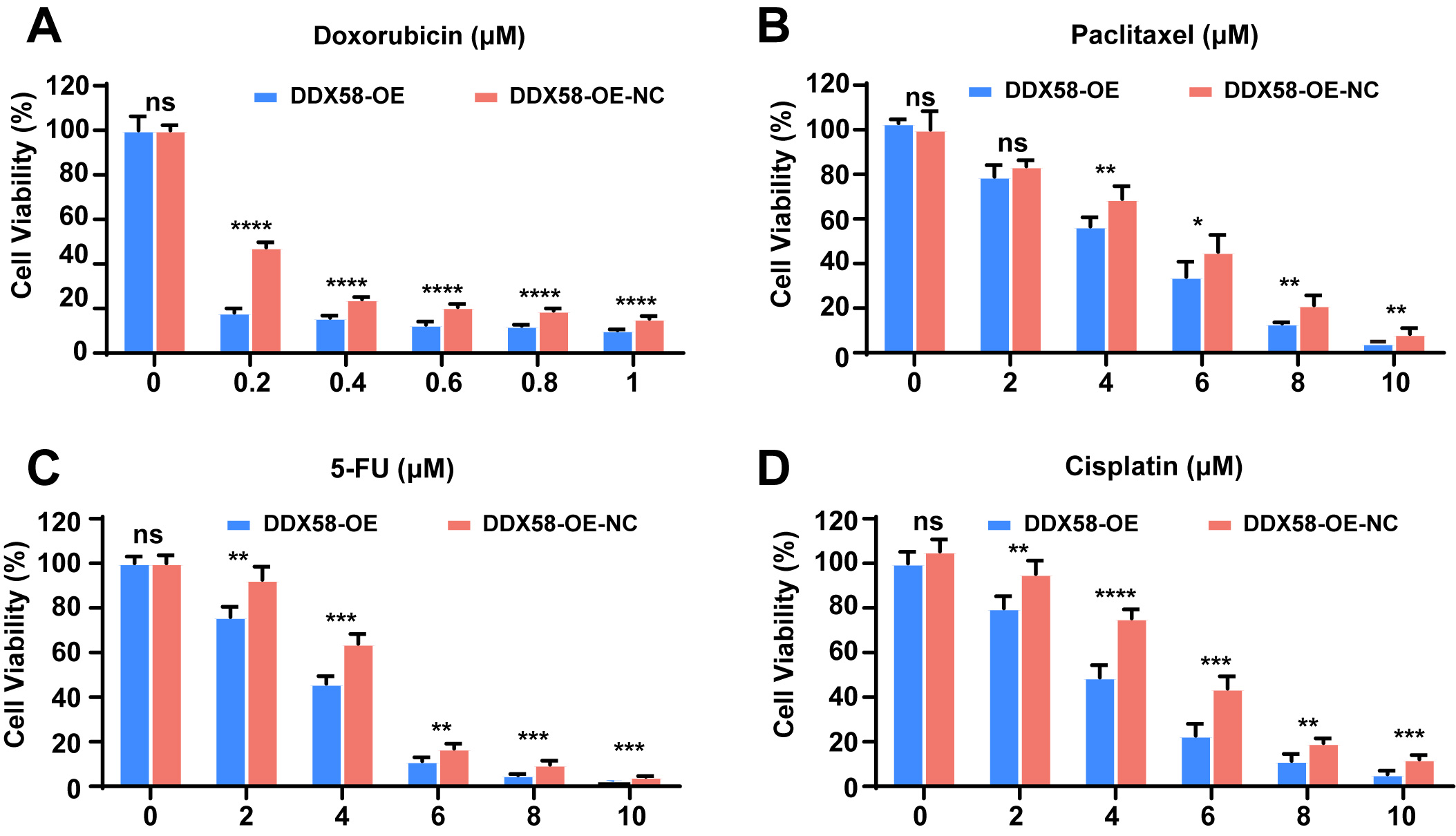

Supplement: Supplementary file 3 [file Image_3.jpg]
