# Supplementary material for: DDX58 deficiency leads to triple negative breast cancer chemotherapy resistance by inhibiting Type I IFN-mediated signalling apoptosis
Source: Front Oncol. 2024 Mar 14;14:1356778. doi: 10.3389/fonc.2024.1356778 (PMC10974639; doi:10.3389/fonc.2024.1356778)
Supplement: Supplementary file 4 [file Table_1.doc]

**Table 1. Baseline clinical characteristics of study subjects**

| **Table1 \| Baseline clinical characteristics of the 196 patients with TNBC** | | | | | | |
| --- | --- | --- | --- | --- | --- | --- |
| **HUAXI data set** | | | | | | |
|  |  |  | **OS** |  | **RFS** |  |
| **Characteristics** | **N%** |  | **p-value** |  | **p-value** |  |
| **WHO Grade** | 196 |  | 0.211 |  | 0.808 |  |
| II | 27 (13.8) |  |  |  |  |  |
| III | 169 (86.2) |  |  |  |  |  |
| **Anatomical stage** | 196 |  | 0.162 |  | 0.059^*^ |  |
| Ⅰ | 50 (25.5) |  |  |  |  |  |
| II | 120 (61.2) |  |  |  |  |  |
| III | 26 (13.3) |  |  |  |  |  |
| **BMI** | 196 |  | 0.855 |  | 0.953 |  |
| >24.0 | 70 (35.7) |  |  |  |  |  |
| ≤ 24.0 | 126(64.3) |  |  |  |  |  |
| **Age** | 196 |  | 0.783 |  | 0.835 |  |
| >50 | 72 (36.7) |  |  |  |  |  |
| ≤50 | 124 (63.3) |  |  |  |  |  |
| **Residence** | 196 |  | 0.496 |  | 0.988 |  |
| Urban | 151 (77) |  |  |  |  |  |
| Rural | 45 (23) |  |  |  |  |  |
| **Menopause at diagnosis** | 196 |  | 0.871 |  | 0.295 |  |
| Yes | 80 (40.8) |  |  |  |  |  |
| No | 116 (59.2) |  |  |  |  |  |
| **Family history of breast cancer** | 196 |  | 0.614 |  | 0.487 |  |
| Yes | 5 (2.6) |  |  |  |  |  |
| No | 191 (97.4) |  |  |  |  |  |
| **T** | 196 |  | 0.015^*^ |  | 0.136 |  |
| 1  2 | 67 (34.2)  121(61.7) |  |  |  |  |  |
| 3-4 | 8 (4.1) |  |  |  |  |  |
| **N** | 196 |  | 0.049^*^ |  | 0.032^*^ |  |
| 0 | 130 (66.3) |  |  |  |  |  |
| 1-3 | 66 (33.7) |  |  |  |  |  |
| **Ki67** | 196 |  | 0.493 |  | 0.337 |  |
| + | 186 (94.9) |  |  |  |  |  |
| - | 9 (4.6) |  |  |  |  |  |
| Unknown | 1 (0.5) |  |  |  |  |  |
| **DDX58 expression** | 196 |  | 0.0066^*^ |  | 0.0197^*^ |  |
| Low expression | 96 (48.5) |  |  |  |  |  |
| High expression | 100 (51.5) |  |  |  |  |  |

* *p* value＜0.05
